# Updated Surveillance Metrics and History of the COVID-19 Pandemic (2020-2023) in the Middle East and North Africa: Longitudinal Trend Analysis

**DOI:** 10.2196/53219

**Published:** 2024-06-12

**Authors:** Alan G Soetikno, Alexander L Lundberg, Egon A Ozer, Scott A Wu, Sarah B Welch, Maryann Mason, Yingxuan Liu, Robert J Havey, Robert L Murphy, Claudia Hawkins, Charles B Moss, Lori Ann Post

**Affiliations:** 1 Feinberg School of Medicine Northwestern University Chicago, IL United States; 2 Buehler Center for Health Policy and Economics Robert J. Havey, MD Institute for Global Health Northwestern University Chicago, IL United States; 3 Department of Emergency Medicine Feinberg School of Medicine Northwestern University Chicago, IL United States; 4 Department of Medicine, Division of Infectious Diseases Feinberg School of Medicine Northwestern University Chicago, IL United States; 5 Center for Pathogen Genomics and Microbial Evolution Robert J. Havey, MD Institute for Global Health Northwestern University Chicago, IL United States; 6 Robert J. Havey, MD Institute for Global Health Northwestern University Chicago, IL United States; 7 Department of Medicine, General Internal Medicine and Geriatrics Feinberg School of Medicine Northwestern University Chicago, IL United States; 8 Center for Global Communicable and Emerging Infectious Diseases Robert J. Havey, MD Institute for Global Health Northwestern University Chicago, IL United States; 9 Institute of Food and Agricultural Sciences University of Florida Gainesville, FL United States

**Keywords:** SARS-CoV-2, COVID-19, Middle East, North Africa, Bahrain, Iran, Iraq, Israel, Jordan, Kuwait, Lebanon, Oman, Qatar, Saudi Arabia, Syria, the United Arab Emirates, Yemen, Algeria, Djibouti, Egypt, Libya, Morocco, Tunisia, pandemic history, COVID-19 transmission, speed, acceleration, deceleration, jerk, dynamic panel, generalized method of moments, Arellano-Bond, 7-day lag

## Abstract

**Background:**

This study updates the COVID-19 pandemic surveillance in the Middle East and North Africa (MENA) we first conducted in 2020 with 2 additional years of data for the region.

**Objective:**

The objective of this study is to determine whether the MENA region meets the criteria for moving from a pandemic to endemic. In doing so, this study considers pandemic trends, dynamic and genomic surveillance methods, and region-specific historical context for the pandemic. These considerations continue through the World Health Organization (WHO) declaration of the end of the public health emergency for the COVID-19 pandemic on May 5, 2023.

**Methods:**

In addition to updates to traditional surveillance data and dynamic panel estimates from the original study by Post et al, this study used data on sequenced SARS-CoV-2 variants from the Global Initiative on Sharing All Influenza Data (GISAID) to identify the appearance and duration of variants of concern. We used Nextclade nomenclature to collect clade designations from sequences and Pangolin nomenclature for lineage designations of SARS-CoV-2. Finally, we conducted a 1-sided *t* test to determine whether regional weekly speed of COVID-19 spread was greater than an outbreak threshold of 10. We ran the test iteratively with 6 months of data from September 4, 2020, to May 12, 2023.

**Results:**

The speed of COVID-19 spread for the region had remained below the outbreak threshold for 7 continuous months by the time of the WHO declaration. Acceleration and jerk were also low and stable. Although the 1- and 7-day persistence coefficients remained statistically significant and positive, the weekly shift parameters suggested the coefficients had most recently turned negative, meaning the clustering effect of new COVID-19 cases became even smaller in the 2 weeks around the WHO declaration. From December 2021 onward, Omicron was the predominant variant of concern in sequenced viral samples. The rolling *t* test of the speed of spread equal to 10 became entirely insignificant from October 2022 onward.

**Conclusions:**

The COVID-19 pandemic had far-reaching effects on MENA, impacting health care systems, economies, and social well-being. Although COVID-19 continues to circulate in the MENA region, the rate of transmission remained well below the threshold of an outbreak for over 1 year ahead of the WHO declaration. COVID-19 is endemic in the region and no longer reaches the threshold of the pandemic definition. Both standard and enhanced surveillance metrics confirm that the pandemic had transitioned to endemic by the time of the WHO declaration.

## Introduction

COVID-19, the disease caused by the virus SARS-CoV-2, was first detected in Wuhan, China, in the fall of 2019 [1-6]. The Middle East and North Africa (MENA) reported their first 9 COVID-19 cases in the United Arab Emirates and Egypt between January 29, 2020, and February 16, 2020 [7]. Our research team first conducted an epidemiological assessment of the pandemic in MENA 1 year into the pandemic [8]. This study provides 2 additional years of updated surveillance and analysis for the region.

We adopt the World Bank's definition of MENA, which is based on economic development and geographical proximity, encompassing Bahrain, Iran, Iraq, Israel, Jordan, Kuwait, Lebanon, Oman, Qatar, Saudi Arabia, Syria, the United Arab Emirates, Yemen, Algeria, Djibouti, Egypt, Libya, Morocco, and Tunisia [9].

The World Health Organization (WHO) and Director-General Ghebreyesus declared the end of COVID-19 as a public health emergency of international concern on May 5, 2023 [10-12] based on the recommendation of the COVID-19 Emergency Committee [12]. We compared how the pandemic, as experienced in the MENA region, progressed before and after the declaration.

Epidemiological terms, such as pandemic, epidemic, outbreak, and endemic, are used to describe the occurrence and spread of diseases [13,14]. The distinctions between these terms lie in their scope, geographic extent, and severity. An epidemic refers to a sudden increase in the number of disease cases in a specific population at the subnational level. If the epidemic spreads across several countries or continents, it becomes a pandemic. An outbreak, on the other hand, describes a sudden increase in a concentrated setting, usually involving a more limited geographic area than an epidemic. Endemic refers to the constant presence of a disease in a particular geographic region or population, with no sudden increases in case volume [15,16]. Field epidemiology defines these terms based on transmission metrics and geographical distribution. All public health surveillance data suffer from incomplete case ascertainment, meaning that not all cases of a disease may be reported or captured in the surveillance system. Despite this limitation, public health surveillance is valuable because it provides a near real-time view of disease trends within a population. Surveillance is crucial for leaders to respond effectively to health threats, implement control measures, and allocate resources where they are needed most. In sum, although the data may not capture every single case, they serve as a critical tool for monitoring and tracking the spread of diseases and health events in a population [17].

Since conducting and publishing our initial research on the pandemic in MENA, much more has been learned about the SARS-CoV-2 virus and its transmission and prevention [18-24]. Additionally, disease control measures and their effects on medical, social, and economic well-being have been further studied in the interval period, with new emphasis on clear guidelines and effective communication strategies [18,19]. In parallel, there was increased focus on risk factors for COVID-19 infection and mortality, ranging from personal risk factors such as age and medical comorbidities to air pollution, climate, and population density [18,25-27]. The vast breadth and depth of new knowledge related to COVID-19 and public health gained in the past 2 years underscore the need for new analysis with updated data and historical context.

Public health surveillance is the “ongoing, systematic collection, analysis, and interpretation of health-related data essential to planning and evaluation of public health practice” [28]. Surveillance explains the burden of disease with transmission and death rates [29-43] and allows us to compare that burden between geographical regions and to understand which regions are most impacted. The impact can be measured through standardized population rates regarding how many people contract or die from a disease.

However, traditional surveillance carries several limitations that this study addressed. Traditional surveillance provides a snapshot of what has already happened [29-43], meaning surveillance is static. In the middle of a burgeoning pandemic, policymakers and public health practitioners also need to understand what is about to happen. Is an outbreak increasing? Will growth switch from linear to exponential? Are more people dying from that particular condition in one place than another? To inform health policy and practice, knowledge of what is about to happen is often more valuable than knowledge of what did happen. To that end, we developed enhanced surveillance metrics that reflect the dynamics of a pandemic and can inform imminent growth and, most importantly, where along the epidemiological outbreak curve a particular region is situated. We also included dynamic metrics about the speed of the pandemic spread at the national, regional, and global levels. We measured how acceleration of speed this week compared with last week, as well as how novel infections last week predicted new cases this week. We can think of the latter measure as the echoing forward of cases. These metrics were tested and validated in prior research [8,44-54].

For the purpose of this study, standard surveillance metrics explain what has already happened in MENA, while enhanced surveillance metrics speak to what is about to happen or where along an epidemiological curve a country may sit. We used both types of metrics to analyze the possible end to the pandemic.

This study had 3 objectives. First, we aimed to measure whether there was a pandemic expansion or contraction in the MENA region at the time the WHO declared the end of the COVID-19 pandemic as a public health emergency of international concern on May 5, 2023. At both the region and country levels, we used advanced surveillance and analytical techniques to describe the status of the pandemic in a 2-week window around the WHO declaration. From a public health perspective, we need to know whether the rate of new COVID-19 cases was increasing, decreasing, or stable from week to week and if any changes in the transmission rate indicated an acceleration or deceleration of the pandemic. Statistical insignificance is significant—it signals an epidemiological “end” to the pandemic if the rate of new cases is 0 (or very low) and stable, meaning the number of new cases is neither accelerating nor decelerating.

Second, we used dynamic and genomic surveillance methods to describe the history of the pandemic in the region and situate the time window around the WHO declaration. We included the ratio of COVID-19 deaths to the number of transmissions as a proxy for the population-level mortality risk from infection. We also included a historical record of genomic surveillance from sequenced viral specimens to identify the appearance and spread of variants of concern in the region.

Third, we aimed to provide historical context for the course of the pandemic in the MENA region. We addressed several questions. How did countries respond to the pandemic? How did the region fare in terms of disease burden? What social, economic, and political factors shaped the course of COVID-19 in the region? This context can provide important lessons for disease prevention and mitigation in future pandemics.

## Methods

### Data Source

This study conducted trend analyses with longitudinal COVID-19 data for the MENA region from 2020 through 2023. Data on COVID-19 transmission and death were sourced from Our World in Data (OWID) [55]. OWID compiles data on COVID-19 cases and mortality from multiple sources, including individual websites, statistical reports, and press releases. The MENA region was defined by the World Bank’s economic and geographical metrics [9], and the data comprised an unbalanced panel of 20 countries and territories from September 4, 2020, to May 12, 2023. Because several countries around the world switched from daily to weekly reports at various points in 2023, we used a cubic spline to interpolate daily new cases and deaths when any country had 4 consecutive periods of non-zero new cases interspersed by 6 days of zero new cases. A cubic spline is a statistical function used to assess the “smoothness” of data points and estimate missing or unclear data on a line given surrounding trends.

To identify the appearance and duration of variants of concern, we used data on sequenced SARS-CoV-2 variants from the Global Initiative on Sharing All Influenza Data (GISAID), which is an effective and trusted online resource for sharing genetic, clinical, and epidemiological COVID-19 data [56-60]. We used Nextclade nomenclature [61] to collect clade designations from sequences and Pangolin nomenclature for lineage designations of SARS-CoV-2 [62,63]. Nextclade nomenclature is an open-source tool for viral genome analysis, mutation identification, clade assignment, and phylogenetic mapping. Pangolin nomenclature is an open-source tool (Phylogenetic Assignment of Named Global Outbreak Lineages) used to track the transmission and spread of SARS-CoV-2 and its lineages. Metadata for the study period were collected on June 22, 2023. To avoid low frequency or potentially erroneous samples, the data set was further filtered to exclude months with fewer than 100 available samples, variant groups with fewer than 5 samples in a month, and variant groups representing less than 0.5% of the total samples in a month. The final data set consisted of 184,386 total samples available on GISAID [56-59].

### Measures

This study provides updates of traditional surveillance data and dynamic panel estimates from the original study by Post et al [8,52,53,64-66]. The “speed” of spread of the pandemic is the rate of new COVID-19 cases per 100,000 population. Novel metrics go beyond speed to add acceleration, jerk, and 1- and 7-day persistence measures. Acceleration is the difference in speed from one week or day to the next. Acceleration identifies whether the number of new cases is increasing (positive acceleration), decreasing (negative acceleration), or at a stable inflection point (zero). “Jerk” is the change in acceleration from one time interval to the next, and its name is adopted from physics nomenclature. A positive jerk can indicate explosive growth in the spread of a disease. Finally, 1- and 7-day persistence measures capture the impact of the 1- and 7-day lag of speed on current speed. These measures capture the echo-forward effect of COVID-19 cases on future cases either 1 or 7 days later. They are derived from coefficient estimates on lagged transmission rates in an Arellano-Bond dynamic panel data model [67]. The model follows the general form of:

y_it_ = ρy_it-1_ + β**X**_it_ + α_i_ + u_it_ **(1)**

where the dependent variable is the rate of COVID-19 transmissions, the independent variables include weekend and recent week indicators, α_i_ denotes country fixed effects, and u_it_ is the idiosyncratic error term. Please see the initial study for more details [8].

We further analyzed the potential “statistical end” to the pandemic with a 1-sided *t* test for whether the mean of speed of spread, defined as the rate of new COVID-19 cases per 100,000 population in a given time period, was equal to or greater than the outbreak threshold of 10. We ran the test on a rolling 6-month window over weekly speed for the region, and we plotted the *P* values from the test over time. All statistical analyses were conducted with the *plm* package (version 2.6-2) in R (version 4.2.1) [64,65].

### Ethical Considerations

This study followed the guidelines of the World Medical Association’s Declaration of Helsinki: Ethical Principles for Medical Research Involving Human Subjects [68,69]. This research relied on publicly available data with no private, identifiable information. Thus, institutional review board review was unsolicited.

## Results

Table 1 presents the dynamic panel estimates for the most recent time window. The Wald test for the regression was significant (*P*<.001), and the Sargan test failed to reject the validity of the overidentification restrictions (*P*≥.99). Although the 1- and 7-day lag coefficients were statistically significant, suggesting a cluster effect in which cases on a given day impact cases 1 day and 7 days later, the coefficients were moderate in magnitude (0.310 and 0.586, respectively). Furthermore, the shift parameters for either of the 2 most recent weeks were both significant and negative, meaning the clustering effect had become smaller—in fact, negative—in the 2 weeks around May 5, 2023.

Static surveillance metrics for the weeks of April 28, 2023, and May 5, 2023, are provided in Table S1 of the Multimedia Appendix 1. Every country had a small number of new COVID-19 cases. The highest rate of new cases per 100,000 population was 8.90 in Qatar for the week of April 28, 2023, still considered a low transmission rate by the Centers for Disease Control and Prevention (CDC) [70]. This rate falls just below the informal outbreak threshold of 10 cases per week per 100,000 population [8,44-54]. Specifically, a “Low” transmission is considered no more than 10 cases per 100,000 people per week. “Moderate” transmission is 10 to 50 cases per 100,000 people per week, and “Substantial” transmission is 50 to 100 cases per 100,000 people per week [70,71]. The weekly transmission rate in Qatar also fell to 0 the following week. For the same week, no other country had a speed greater than 2.

Comparisons in Table S1 (Multimedia Appendix 1) demonstrate little to no change in surveillance metrics before and after the WHO declared an end to the COVID-19 emergency. Without question, Iran and Israel had the most cases of COVID-19 transmissions and deaths, but this rank is a function of population size. Thus, a better measure is the number of COVID-19 cases and deaths per 100,000 population. Moreover, death is often a better proxy for the state of an outbreak than transmissions because deaths are less likely to be undercounted [72]. Undercounting may be due to poor public health infrastructure, home antigen testing, or a dearth of polymerase chain reaction (PCR) testing or other resources. Iran reported 0.02 deaths per 100,000 population. When we controlled for a risk of death given the number of COVID-19 transmissions, we found that Egypt had the highest conditional death rate of 0.05 deaths per case. The next highest rate was 0.03 deaths per case in both Algeria and Tunisia.

Table 2 contains enhanced dynamic surveillance metrics for the 2 weeks before and after May 5, 2023. Speed of spread was low for every country except Qatar in the week of April 28, 2023, and acceleration was 0 or negative for almost every country. During this time, Qatar still remained well below the outbreak threshold (see Table S1 in Multimedia Appendix 1). Although positive, acceleration was small. The 7-day persistence effect on speed of spread was also very small in magnitude for the week of April 28, 2023, in every country, and the persistence effect fell to 0 or negative for every country the following week. These metrics suggest the pandemic may have indeed ended for the region. We note that the figures in Table 2 are not calculated as day-over-day averages across the week, as they are in Table S1 in Multimedia Appendix 1. Thus, the magnitudes of speed may not exactly match those in Table S1 in Multimedia Appendix 1.

Table 3 compares the 7-day persistence effect on speed for the 5 countries with the highest persistence for the weeks of April 28, 2023, and May 5, 2023. In each case, the effect had become either 0 or negative by the second week. Again, these metrics indicate that COVID-19 was well controlled in the region overall.

**Figure 1:**
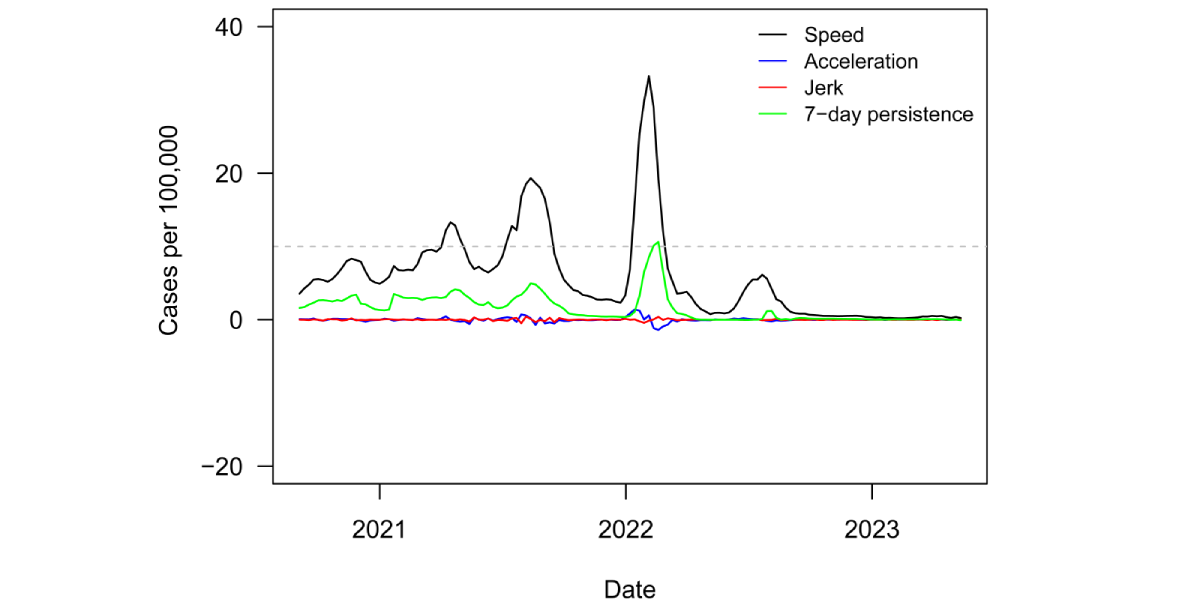
Timeline of speed, acceleration, jerk, and 7-day persistence in the Middle East and North Africa from 2020 to 2023.

Figure 1 plots the regional speed of spread, acceleration, jerk, and 7-day persistence metrics from September 4, 2020, to May 12, 2023. The dashed grey line denotes the informal CDC outbreak threshold of speed equal to 10. The region experienced 3 outbreaks over the course of the pandemic. The first was brief, reaching a peak speed of only 13 in April 2021. The second saw a peak speed of 19 in August 2021. However, these 2 outbreaks can largely be considered 1, as speed dipped only slightly below the outbreak threshold of 10 between them. The third outbreak was the largest, with a peak speed of 33 in February 2022.

**Figure 2:**
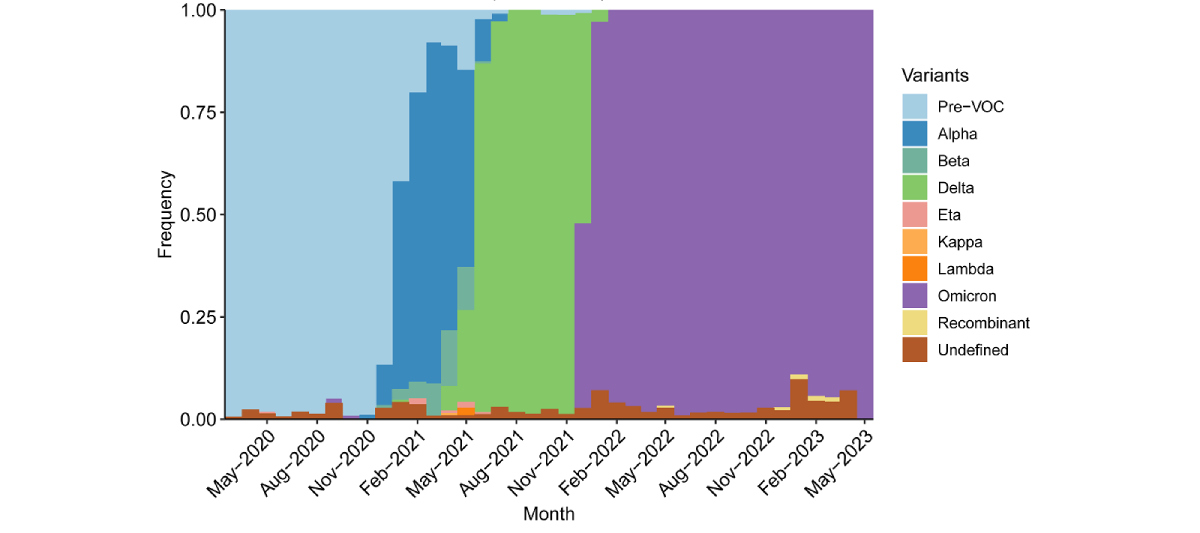
Variant of concern (VOC) groups as a proportion of all sequenced SARS-CoV-2 specimens over time in the Middle East and North Africa from May 2020 through May 2023.

Figure 2 plots variant groups as a proportion of all viral specimens collected and sequenced in the region (and made available through GISAID) each month. The first 1 to 2 outbreaks referenced in the previous paragraph occurred just around the appearance of the Delta variant. The last outbreak was driven by the Omicron variant. MENA, like much of the rest of the world, saw a surge in cases amid the heightened transmissibility of Omicron [73]. Still, the outbreak was much smaller than in several other regions of the world, such as North America, Europe, and East Asia and the Pacific, which each saw peak speeds of over 200 amid Omicron outbreaks.

Another potential indication of the end to the pandemic was the continued dominance of the Omicron variant. Although the region saw a mixture of the ancestral, Alpha, Beta, and Delta variants prior to the arrival of Omicron in November 2021, viral sequences have almost exclusively returned as Omicron and its subvariants ever since.

**Figure 3:**
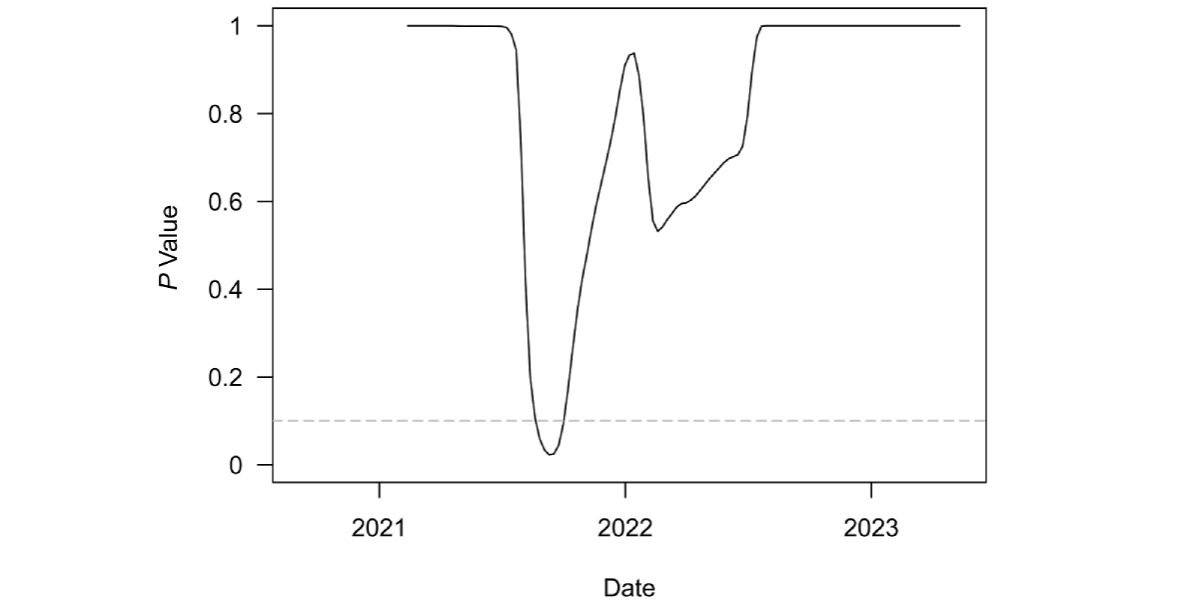
Rolling *t* test of weekly speed equal to 10 over a 6-month window in the Middle East and North Africa from 2020 through 2023.

Figure 3 plots the *P* values from a series of 1-sided *t* tests to determine whether speed for the region was equal to or greater than the threshold outbreak of 10. These tests were conducted on a rolling 6-month window of weekly regional speed. The dashed grey line denotes the least restrictive conventional significance level threshold of α=.10. The only time the test rejected the null hypothesis in favor of the alternative occurred around the 6-month period ending in mid-August 2021. This period marked the extended, intermittent outbreak driven by (presumably) the Delta variant. From then on, the test failed to reject the null hypothesis, although *P* values did drop again somewhat around the later Omicron outbreak. The test statistic became consistently insignificant from approximately October 2022 onward. This more recent lack of statistical significance is consistent with the end of the pandemic in the region, as the test clearly failed to reject the null hypothesis of outbreak threshold speed.

With the historical context of enhanced surveillance metrics, the MENA region appeared to be at the end stage of the pandemic. Speed had not been this low for this long since the start of the pandemic. Furthermore, speed remained well below outbreak status for over 1 year ahead of the WHO declaration.

**Figure 4:**
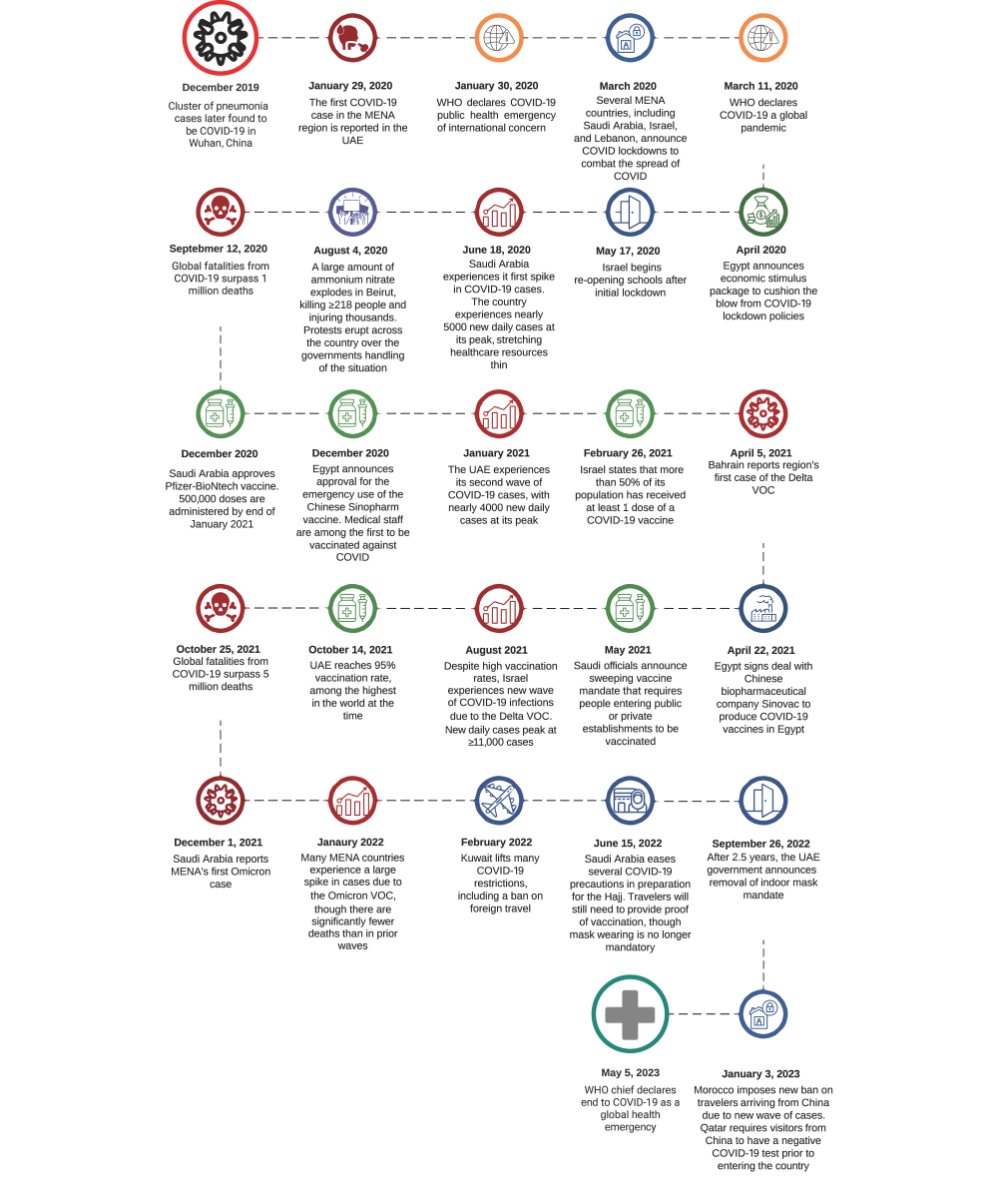
Timeline of the COVID-19 pandemic in the Middle East and North Africa (MENA). VOC: variant of concern; WHO: World Health Organization.

Figure 4 provides a timeline of the onset of COVID-19 in MENA as well as vaccination programs and major events that likely created additional challenges to disease control, such as the arrival of new variants of concern.

**Table 1:** Arellano-Bond dynamic panel data modeling from Equation (1) of the number of daily infections reported by country, April 28, 2023, through May 12, 2023.^a,b^

Variable	Value^c^	*P* value
1-day persistence coefficient	0.310	<.001
7-day persistence coefficient	0.586	<.001
Shift parameter week of April 28, 2023	–0.752	<.001
Shift parameter week of May 5, 2023	–0.304	<.001
Weekend	0.300	.17

^a^Wald test: χ^2^_6_=12567.77, *P*<2.22^–16^.

^b^Sargan: χ^2^_540_=13, *P*≥.99.

^c^Contains estimates from the model in Equation (1).

**Table 2:** Novel surveillance metrics in Middle East and North Africa (MENA) for the weeks of April 28, 2023, and May 5, 2023.

Country	April 28, 2023				May 5, 2023			
	Speed^a^	Acceleration^b^	Jerk^c^	7-day persistence effect on speed^d^	Speed^a^	Acceleration^b^	Jerk^c^	7-day persistence effect on speed^d^
Algeria	0.02	0	0	0	0.02	0	0	0
Egypt	0	0	0	0	0	0	0	0
Iran	0.22	0	0.06	0.04	0.20	0	0	–0.04
Israel	2.39	0.05	–0.09	0.19	1.84	–0.12	–0.05	–0.40
Kuwait	0.20	0	0	0.03	0.16	0	0	–0.03
Lebanon	1.17	0	0.27	0.10	1.08	0.67	0.84	–0.19
Libya	0	0	0	0	0	0	0	0
Morocco	0.13	0	0	0.01	0.14	0	0	–0.02
Oman	0	0	0	0	0	0	0	0
Qatar	16.52	1.27	–0.25	0	6.13	–1.27	–0.82	–2.73
Saudi Arabia	0.50	-0.04	0	0.04	0	-0.05	0.01	–0.08
Tunisia	2.09	0.06	–0.04	0.12	1.52	–0.13	0	–0.35
United Arab Emirates	2.21	0.01	–0.08	0.15	2.08	–0.06	–0.01	–0.37

^a^New COVID-19 cases per 100,000 population.

^b^Difference in speed from one week to the next.

^c^Change in acceleration from one week to the next.

^d^The impact of the 1- and 7-day lags of speed on current speed.

**Table 3:** Top 5 countries ranked by 7-day persistence in the Middle East and North Africa (MENA) for the weeks of April 28, 2023, and May 5, 2023.

Week and country		7-day persistence^a^
**Week of April 28, 2023**		
	Israel	0.19
	United Arab Emirates	0.15
	Tunisia	0.12
	Lebanon	0.10
	Saudi Arabia	0.04
**Week of May 5, 2023**		
	Libya	0
	Oman	0
	Egypt	0
	Algeria	0
	Morocco	-0.02

^a^7-day persistence was estimated using Equation (1).

## Discussion

### Principal Findings

The principal finding to the stated objective of this study is that the MENA region meets the 3 criteria for moving from a pandemic to an endemic. First, the transmission rates before and after the WHO declaration of the end of the COVID-19 public health emergency of international concern on May 5, 2023, were well below the threshold of an outbreak. Second, the transmission and death rates approached 0 and remained extremely small and stable. Finally, the pandemic in MENA is no longer propagating forward. Our findings therefore signal that COVID-19 has changed from a pandemic to an endemic in MENA. Nonetheless, COVID-19 had profound effects on the region’s economy, political, and public health institutions since the pandemic began in spring 2020.

The MENA region saw several waves of infection over the course of the pandemic, with significant spikes driven by new SARS-CoV-2 variants and the rollback of COVID-19 restrictions. The region saw significant disparities in how effectively countries were able to enforce quarantine rules and contain the virus. During an initial wave in 2020, MENA countries with the economic, social, and political resources to institute quarantines were quick to respond to the pandemic and had substantially lower infection and fatality rates compared with countries in Europe and North America. This response allowed them to reopen their economies quickly. MENA governments similarly responded to later waves by reinstituting quarantine rules in focused areas. For example, Saudi Arabia, Kuwait, and Qatar each saw the number of COVID-19 cases drastically increase in May 2020 after restrictions were eased for Ramadan. These governments quickly reinforced strict lockdowns to control the spread of the virus [74].

Other MENA countries, which were already struggling with economic and sociopolitical instability, faced heightened crises as COVID-19 strained their already fragile societies. In Syria, which has grappled with internal conflict since 2011, COVID-19 created a health care catastrophe as the country’s already strained health care system was pushed to the breaking point. According to the WHO, only 50% of hospitals in Syria were fully functioning, and 25% were partially functioning at the start of the pandemic due to shortages of staff and medical equipment from years of war [75]. Refugee camps, which lacked proper medical staff and equipment, created grounds for the virus to spread quickly [76]. Yemen, which has dealt with a long and protracted civil conflict, faced its own crisis in the pandemic. Trade restrictions due to COVID-19 led to more than 50% of Yemen’s population facing food insecurity and hunger [77-79].

In economic impacts, the MENA region saw a 3.5% decline in gross domestic product (GDP) in 2020 driven by COVID-19 containment measures. However, as vaccines became more prevalent and countries began easing COVID restrictions, economies rebounded, with GDP growth of 4.5% in 2021 [80]. Some countries, such as Saudi Arabia, Kuwait, and the United Arab Emirates, also faced a secondary economic shock from a sharp decline in oil prices in 2020 [81].

Many countries in the region approved large fiscal packages to support struggling economic sectors. These packages typically included direct cash support for affected workers, tax relief for individuals and corporations, investment in strained health care systems, and economic investment in specific sectors. Saudi Arabia announced a SR 70 billion (US $18.6 billion; 2.7% of GDP) private support package, which suspended government tax payments and provided additional liquidity to the private sector. Kuwait allocated KD 500 million (US $1.6 billion; 1.5% of GDP) toward economic stimulus, which included the provision of full unemployment benefits for Kuwaiti nationals [81]. Many MENA countries began the pandemic with underfunded health care systems and had to drastically increase their health care expenditure and invest in personal protective equipment, ventilators, and other medical supplies. Egypt allocated £5 billion (US $103 million) toward the purchase of necessary medical supplies and funds for additional medical staff [81]. Overall, these health and social welfare packages required MENA countries to borrow substantial amounts of money, leading to increases in government debt [82].

Given prior experience with the Middle East respiratory syndrome (MERS), many countries in the region were quickly able to enact containment strategies. The backbone of these strategies consisted of masking, border closures, contact tracing, social distancing, quarantines, and lockdowns. However, there were significant disparities in how effective these measures were implemented given the large differences in economic resources, health care infrastructure, and the political stability between countries. The United Arab Emirates was one of the first countries to issue a COVID-19 alert before the WHO declared COVID-19 a public health emergency. Saudi Arabia, a deeply religious country, barred Muslims from conducting prayers inside mosques. Pilgrimages to holy sites such as Mecca and Medina were banned for foreigners [83]. Additionally, the United Arab Emirates and Saudi Arabia levied fines against any individuals or businesses that failed to adhere to mask mandate policies [84]. Saudi Arabia also built makeshift hospitals to increase their health care capacity and accommodate surges in COVID-19 cases [85]. Israel’s Ministry of Health implemented a containment strategy focused on early travel restrictions to countries reporting COVID-19 cases, and 14-day self-quarantine measures were imposed on anyone arriving from abroad [86]. More extensive lockdown measures were ultimately implemented, including the closure of schools, universities, and all nonessential businesses [87]. Another cornerstone of Israel’s containment strategy was the use of its extensive health care resources to test and quarantine patients at scale [88]. Israel also utilized mobile apps and spatial data collected from mobile phones to effectively perform contact tracing [88,89].

Some countries in the region lacked the health care resources to effectively fight the spread of the virus. At the start of the pandemic, Morocco had a hospital bed capacity of 1.1 beds for 1000 people, among the lowest in MENA [90]. Morocco therefore adopted an early, strict containment strategy to prevent straining their limited health care system. Policies included suspension of public events, suspension of international travel, and restrictions on intercity travel [18,21,25,90,91]. Additionally, the government created a “COVID-19 Fund,” which provided funding to increase hospital beds and intensive care unit capacity, purchase personal protective equipment, and increase testing capabilities [92]. Morocco experienced one of the lowest fatality rates in the region during the initial wave of infections [92].

Yemen faced difficulties implementing a comprehensive containment strategy given the violent civil conflict facing the country. Health infrastructure lacked the capability to properly test patients and track the virus [93]. In parts of the country controlled by the Houthi faction, almost no COVID-19 restrictions were implemented [81,94].

As the pandemic continued, countries eventually relaxed their restrictions to stimulate the economy and only reinstated lockdowns when COVID-19 cases began to spike [22,95]. MENA countries focused on achieving herd immunity through widespread vaccination campaigns. The Pfizer-BioNTech vaccine was the first vaccine approved in Saudi Arabia in mid-December 2020, with millions of vaccine doses being administered throughout the pandemic [96]. In July 2021, Saudia Arabia was also one of the world’s first countries to implement a large-scale vaccine mandate to combat a new surge in cases from the COVID-19 Delta variant. This mandate required proof of vaccination to enter public and private institutions, including schools, shops, malls, public transportation, and more [97]. The United Arab Emirates also mandated vaccinations for all people attending live events such as sports events and art exhibitions. Attendees also had to provide a negative polymerase chain reaction (PCR) test within 48 hours of the event [98]. Egypt announced that COVID-19 vaccinations were mandatory for people over the age of 18 years. The country banned all nonvaccinated people from entering public, governmental, and educational buildings to further incentivize vaccination [99]. In June 2021, Egypt also signed an agreement with Chinese vaccine manufacturer SinoVac to locally produce the company’s inactivated vaccine, CoronaVac [100,101].

There were large disparities in access to vaccines, with richer nations such as the United Arab Emirates and Saudi Arabia able to vaccinate their population much faster than less-resourced countries. For example, by early December 2021, the United Arab Emirates had one of the world’s highest vaccination rates at 90% of the population, while Yemen had just reached a rate of 1% [23,82].

One unique issue facing countries in the MENA region was the need for halal-certified vaccines. Many countries in the region have significant Muslim populations [102]. Islamic law requires that Muslims avoid using vaccines manufactured with certain types of ingredients (such as pork products) [102]. To avoid potential vaccine hesitancy, religious leaders in several MENA countries, including the United Arab Emirates and Egypt, declared vaccines halal and encouraged citizens to get vaccinated to prevent further spread of the virus [103].

### Limitations

Limitations of our data analysis and resulting manuscript include the following. COVID-19 data had become less frequently reported around the world by the time the WHO declared an end to the COVID-19 Emergency of International Concern [104]. Additionally, more people began to use at-home tests as the pandemic evolved [105]. Because the enhanced surveillance metrics of speed, acceleration, jerk, and 7-day persistence are based on rates, not total counts, statistical bias caused by countries dropping in or out of the sample is mitigated, but to the extent that a nonincluded country is unrepresentative of the region in disease burden, the omission of a country or territory can still influence historical data comparisons. Viral specimen tests for variants of concern in GISAID are also dependent on testing and sequencing capacity, which varied by country across the region.

### Conclusion

Overall, the COVID-19 pandemic had far-reaching effects on MENA, impacting health care systems, economies, and social well-being. Although the region fared better in disease burden than several others, many countries in the region continue to face challenges due to limited economic and health care resources [48,106-108]. Although COVID-19 continues to circulate in MENA, the rate of transmission remained well below the threshold of an outbreak for over 1 year ahead of the WHO declaration.

The concern about potential resurgences of the SARS-CoV-2 virus is valid [20,109-111]. As long as COVID-19 continues to spread and mutate, the possibility of new variants emerging remains. Variants could potentially be more transmissible, resistant to vaccines, or cause more severe illness. For example, 4 months after the WHO declared the end to the public health emergency, Omicron has further mutated into Omicron EG.5, incorrectly referred to as Eris and sensationalized in the media [112]. There is no evidence that this subclade results in more severe disease or death [113]. This underscores the importance, however, of continued vigilance, vaccination efforts, and global cooperation to control the spread of the virus [19,24,26,27,51,114-116].

Our findings underscore the importance of the following efforts on a country and regional basis: government investment in epidemiological surveillance and preparedness for future pandemics and public health emergencies; identification of barriers to access of key public health resources including care (including acute and preventative care [ie, vaccines]), education, and material goods and financial support; additional crisis management preparations in the social and economic spheres given their intersectionality with public health; regular assessment of social attitudes toward health crises, including both ongoing and potential future emergencies.

## Appendix

Multimedia Appendix 1 Static surveillance metrics for the weeks of Apr 28, 2023 (Pre-Declaration) and May 5, 2023 (Post-Declaration).

## Abbreviations

CDC: Centers for Disease Control and Prevention
GDP: gross domestic product
GISAID: Global Initiative on Sharing All Influenza Data
MENA: Middle East and North Africa
MERS: Middle East respiratory syndrome
OWID: Our World in Data
PCR: polymerase chain reaction
WHO: World Health Organization

## Data Availability

All data in this study are unrestricted and publicly available in the Our World in Data (OWID) and Global Initiative on Sharing All Influenza Data (GISAID) repositories described in the Methods section [55,56].

## References

[1] Muralidar S, Ambi SV, Sekaran S, Krishnan UM (2020). The emergence of COVID-19 as a global pandemic: Understanding the epidemiology, immune response and potential therapeutic targets of SARS-CoV-2. Biochimie.

[2] Sharma A, Ahmad Farouk I, Lal SK (2021). COVID-19: a review on the novel coronavirus disease evolution, transmission, detection, control and prevention. Viruses.

[3] Chilamakuri R, Agarwal S (2021). COVID-19: characteristics and therapeutics. Cells.

[4] Hu B, Guo H, Zhou P, Shi Z (2021). Characteristics of SARS-CoV-2 and COVID-19. Nat Rev Microbiol.

[5] Seyed Hosseini E, Riahi Kashani N, Nikzad H, Azadbakht J, Hassani Bafrani H, Haddad Kashani H (2020). The novel coronavirus disease-2019 (COVID-19): Mechanism of action, detection and recent therapeutic strategies. Virology.

[6] Núñez-Delgado A, Bontempi E, Coccia M, Kumar M, Farkas K, Domingo JL (2021). SARS-CoV-2 and other pathogenic microorganisms in the environment. Environ Res.

[7] (2020). Update on COVID-19 in the Eastern Mediterranean Region. World Health Organization.

[8] Post L, Marogi E, Moss CB, Murphy RL, Ison MG, Achenbach CJ, Resnick D, Singh L, White J, Boctor MJ, Welch SB, Oehmke JF (2021). SARS-CoV-2 surveillance in the Middle East and North Africa: longitudinal trend analysis. J Med Internet Res.

[9] Middle East and North Africa. The World Bank.

[10] Smith-Schoenwalder C (2023). When Does the COVID-19 Pandemic End?. U.S. News & World Report.

[11] Burki T (2023). WHO ends the COVID-19 public health emergency. The Lancet Respiratory Medicine.

[12] (2023). WHO chief declares end to COVID-19 as a global health emergency. United Nations.

[13] Lesson 1: Introduction to Epidemiology. Centers for Disease Control and Prevention.

[14] (2021). Epidemic, Endemic, Pandemic: What are the Differences?. Columbia University Mailman School of Public Health.

[15] The Lancet Infectious Diseases (2022). Transitioning to endemicity with COVID-19 research. The Lancet Infectious Diseases.

[16] Britto D, Delorme J, Miller B, Sleat D, Summers H, Wain R (2021). Pandemic to Endemic: The Race Against Time. Tony Blair Institute for Global Change.

[17] (2013). Analyze and interpret surveillance data. Centers for Disease Control and Prevention.

[18] Coccia M (2023). Sources, diffusion and prediction in COVID-19 pandemic: lessons learned to face next health emergency. AIMS Public Health.

[19] Coccia M (2023). Effects of strict containment policies on COVID-19 pandemic crisis: lessons to cope with next pandemic impacts. Environ Sci Pollut Res Int.

[20] Coccia M (2022). COVID-19 vaccination is not a sufficient public policy to face crisis management of next pandemic threats. Public Organiz Rev.

[21] Benati I, Coccia M (2022). Effective contact tracing system minimizes COVID-19 related infections and deaths: policy Lessons to Reduce the impact of future pandemic diseases. Journal of Public Administration and Governance.

[22] Coccia M (2022). Preparedness of countries to face COVID-19 pandemic crisis: Strategic positioning and factors supporting effective strategies of prevention of pandemic threats. Environ Res.

[23] Benati I, Coccia M (2022). Global analysis of timely COVID-19 vaccinations: improving governance to reinforce response policies for pandemic crises. IJHG.

[24] Coccia M (2022). COVID-19 pandemic over 2020 (withlockdowns) and 2021 (with vaccinations): similar effects for seasonality and environmental factors. Environ Res.

[25] Akan AP, Coccia M (2023). Transmission of COVID-19 in cities with weather conditions of high air humidity: lessons learned from Turkish Black Sea region to face next pandemic crisis. COVID.

[26] Bontempi E, Coccia M, Vergalli S, Zanoletti A (2021). Can commercial trade represent the main indicator of the COVID-19 diffusion due to human-to-human interactions? A comparative analysis between Italy, France, and Spain. Environ Res.

[27] Coccia M (2021). Pandemic Prevention: Lessons from COVID-19. Encyclopedia.

[28] Hong R, Walker R, Hovan G, Henry L, Pescatore R (2020). The power of public health surveillance. Dela J Public Health.

[29] Teutsch SM, Teutsch SM, Churchill RE (2000). Considerations in Planning a Surveillance System. Principles and Practice of Public Health Surveillance.

[30] Teutsch SM, Lee LM, Teutsch SM, Thacker SB, St. Louis ME (2010). Considerations in Planning a Surveillance System. Principles & Practice of Public Health Surveillance (3rd edn).

[31] Teutsch SM, Thacker SB (1995). Planning a public health surveillance system. Epidemiol Bull.

[32] Thacker S, Berkelman RL (1988). Public health surveillance in the United States. Epidemiol Rev.

[33] Klaucke DN, Buehler JW, Thacker SB, Parrish RG, Trowbridge FL, Berkelman RL, The Surveillance Coordination Group (1988). Guidelines for evaluating surveillance systems. Morbidity and Mortality Weekly Report.

[34] Lee LM, Thacker SB (2011). Public health surveillance and knowing about health in the context of growing sources of health data. Am J Prev Med.

[35] Davis AM, Dunet DO, Keaton R, Snider DE, Sosin DM, Stroup DF, Teutsch SM, Thacker SB, Truman BI, The Centers for Disease Control and Prevention (U.S.) (1996). CDC guidelines : improving the quality. Centers for Disease Control and Prevention.

[36] Thacker S, Stroup DF (1994). Future directions for comprehensive public health surveillance and health information systems in the United States. Am J Epidemiol.

[37] Nsubuga P, White ME, Thacker SB, Anderson MA, Blount SB, Broome CV, Chiller TM, Espitia V, Imtiaz R, Sosin D, Stroup DF, Tauxe RV, Vijayaraghavan M, Trostle M, Jamison DT, Breman JG, Measham AR, Alleyne G, Claeson M, Evans DB, Jha P, Mills A, Musgrove P (2006). Public Health Surveillance: A Tool for Targeting and Monitoring Interventions. Disease Control Priorities in Developing Countries. 2nd edition.

[38] Lee (ed.) LM, Teutsch (ed.) SM, Thacker (ed.) SB, St. Louis (ed.) ME (2010). Principles & Practice of Public Health Surveillance.

[39] Thacker SB, Stroup DF, Rothenberg RB (1995). Public health surveillance for chronic conditions: a scientific basis for decisions. Stat Med.

[40] Perry HN, McDonnell SM, Alemu W, Nsubuga P, Chungong S, Otten MW, Lusamba-dikassa PS, Thacker SB (2007). Planning an integrated disease surveillance and response system: a matrix of skills and activities. BMC Med.

[41] Koo D, Thacker SB (2010). In snow's footsteps: Commentary on shoe-leather and applied epidemiology. Am J Epidemiol.

[42] Romaguera RA, German RR, Klaucke DN, Teutsch SM, Churchill RE (2000). Evaluating Public Health Surveillance. Principles & Practice of Public Health Surveillance.

[43] Pappaioanou M, Malison M, Wilkins K, Otto B, Goodman RA, Churchill R, White M, Thacker SB (2003). Strengthening capacity in developing countries for evidence-based public health: the data for decision-making project. Soc Sci Med.

[44] Post L, Boctor MJ, Issa TZ, Moss CB, Murphy RL, Achenbach CJ, Ison MG, Resnick D, Singh L, White J, Welch SB, Oehmke JF (2021). SARS-CoV-2 surveillance system in Canada: longitudinal trend analysis. JMIR Public Health Surveill.

[45] Post L, Culler K, Moss CB, Murphy RL, Achenbach CJ, Ison MG, Resnick D, Singh LN, White J, Boctor MJ, Welch SB, Oehmke JF (2021). Surveillance of the second wave of COVID-19 in Europe: longitudinal trend analyses. JMIR Public Health Surveill.

[46] Post L, Ohiomoba RO, Maras A, Watts SJ, Moss CB, Murphy RL, Ison MG, Achenbach CJ, Resnick D, Singh LN, White J, Chaudhury AS, Boctor MJ, Welch SB, Oehmke JF (2021). Latin America and the Caribbean SARS-CoV-2 surveillance: longitudinal trend analysis. JMIR Public Health Surveill.

[47] Post LA, Argaw ST, Jones C, Moss CB, Resnick D, Singh LN, Murphy RL, Achenbach CJ, White J, Issa TZ, Boctor MJ, Oehmke JF (2020). A SARS-CoV-2 surveillance system in Sub-Saharan Africa: modeling study for persistence and transmission to inform policy. J Med Internet Res.

[48] Post LA, Benishay ET, Moss CB, Murphy RL, Achenbach CJ, Ison MG, Resnick D, Singh LN, White J, Chaudhury AS, Boctor MJ, Welch SB, Oehmke JF (2021). Surveillance metrics of SARS-CoV-2 transmission in Central Asia: longitudinal trend analysis. J Med Internet Res.

[49] Post LA, Issa TZ, Boctor MJ, Moss CB, Murphy RL, Ison MG, Achenbach CJ, Resnick D, Singh LN, White J, Faber JMM, Culler K, Brandt CA, Oehmke JF (2020). Dynamic public health surveillance to track and mitigate the US COVID-19 epidemic: longitudinal trend analysis study. J Med Internet Res.

[50] Post LA, Lin JS, Moss CB, Murphy RL, Ison MG, Achenbach CJ, Resnick D, Singh LN, White J, Boctor MJ, Welch SB, Oehmke JF (2021). SARS-CoV-2 wave two surveillance in East Asia and the Pacific: longitudinal trend analysis. J Med Internet Res.

[51] Post LA, Lorenzo-Redondo R (2022). Omicron: fewer adverse outcomes come with new dangers. Lancet.

[52] Oehmke JF, Moss CB, Singh LN, Oehmke TB, Post LA (2020). Dynamic panel surveillance of COVID-19 transmission in the United States to inform health policy: observational statistical study. J Med Internet Res.

[53] Oehmke JF, Oehmke TB, Singh LN, Post LA (2020). Dynamic panel estimate-based health surveillance of SARS-CoV-2 infection rates to inform public health policy: model development and validation. J Med Internet Res.

[54] Oehmke TB, Post LA, Moss CB, Issa TZ, Boctor MJ, Welch SB, Oehmke JF (2021). Dynamic panel data modeling and surveillance of COVID-19 in metropolitan areas in the United States: longitudinal trend analysis. J Med Internet Res.

[55] Mathieu E, Ritchie H, Rodés-Guirao L, Appel C, Gavrilov D, Giattino C, Hasell J, Macdonald B, Dattani S, Beltekian D, Ortiz-Ospina E, Roser M (2020). Coronavirus pandemic (COVID-19). Our World in Data.

[56] Global Initiative on Sharing All Influenza Data (GISAID).

[57] Khare S, Gurry C, Freitas L, Schultz MB, Bach G, Diallo A, Akite N, Ho J, Lee RTC, Yeo W, Maurer-Stroh S, GISAID Core Curation Team (2021). GISAID's role in pandemic response. China CDC Wkly.

[58] Shu Y, McCauley J (2017). GISAID: Global initiative on sharing all influenza data - from vision to reality. Euro Surveill.

[59] Nasereddin A, Golan Berman H, Wolf DG, Oiknine-Djian E, Adar S (2022). Identification of SARS-CoV-2 variants of concern using amplicon next-generation sequencing. Microbiol Spectr.

[60] Raza S, Schwartz B (2023). Constructing a disease database and using natural language processing to capture and standardize free text clinical information. Sci Rep.

[61] Huddleston J, Hadfield J, Sibley T, Lee J, Fay K, Ilcisin M, Harkins E, Bedford T, Neher R, Hodcroft E (2021). Augur: a bioinformatics toolkit for phylogenetic analyses of human pathogens. J Open Source Softw.

[62] Rambaut A, Holmes EC, O'Toole Á, Hill V, McCrone JT, Ruis C, du Plessis L, Pybus OG (2020). A dynamic nomenclature proposal for SARS-CoV-2 lineages to assist genomic epidemiology. Nat Microbiol.

[63] O'Toole Á, Scher E, Underwood A, Jackson B, Hill V, McCrone JT, Colquhoun R, Ruis C, Abu-Dahab K, Taylor B, Yeats C, du Plessis L, Maloney D, Medd N, Attwood SW, Aanensen DM, Holmes EC, Pybus OG, Rambaut A (2021). Assignment of epidemiological lineages in an emerging pandemic using the pangolin tool. Virus Evol.

[64] Croissant Y, Millo G (2008). Panel data econometrics in R: The plm Package. Journal of Statistical Software.

[65] R Core Team (2023). R: A Language and Environment for Statistical Computing. R Foundation for Statistical Computing.

[66] Hansen LP (1982). Large sample properties of generalized method of moments estimators. Econometrica.

[67] Arellano M, Bond S (1991). Some tests of specification for panel data: Monte Carlo evidence and an application to employment equations. The Review of Economic Studies.

[68] (2022). WMA Declaration of Helsinki - Ethical Principles for Medical Research Involving Human Subjects. World Medical Association.

[69] Hu (ed.) M (2022). Pandemic Surveillance: Privacy, Security, and Data Ethics.

[70] Levenson E, Firger J (2021). What the CDC’s ‘substantial’ and ‘high’ levels of Covid-19 transmission actually mean. CNN.

[71] Christie A, Brooks JT, Hicks LA, Sauber-Schatz EK, Yoder JS, Honein MA, CDC COVID-19 Response Team (2021). Guidance for implementing COVID-19 prevention strategies in the context of varying community transmission levels and vaccination coverage. MMWR Morb Mortal Wkly Rep.

[72] Stoto M (1992). Public health assessment in the 1990s. Annu Rev Public Health.

[73] Lundberg AL, Lorenzo-Redondo R, Ozer EA, Hawkins CA, Hultquist JF, Welch SB, Prasad PV, Oehmke JF, Achenbach CJ, Murphy RL, White JI, Havey RJ, Post LA (2022). Has Omicron changed the evolution of the pandemic?. JMIR Public Health Surveill.

[74] Dadouch S (2020). Saudi Arabia, other gulf states reimpose strict measures after coronavirus cases spike during Ramadan. The Washington Post.

[75] (2020). Syria: Lack of adequate COVID-19 response puts thousands of lives at risk. Amnesty International.

[76] Conway D (2020). Syria: Inside a refugee camp where Covid is spreading. BBC.

[77] Nasser A (2020). War and COVID-19 in Yemen. Human Rights Watch.

[78] Stone R (2020). Yemen was facing the world's worst humanitarian crisis. Then the coronavirus hit. Science.

[79] Rahmat Z, Islam Z, Mohanan P, Kokash DM, Essar MY, Hasan MM, Hashim HT, Basalilah AFM (2022). Food Insecurity during COVID-19 in Yemen. Am J Trop Med Hyg.

[80] The World Bank.

[81] Policy responses to COVID-19. International Monetary Fund.

[82] Overview: Middle East and North Africa. The World Bank.

[83] Gehrke L (2020). Saudi Arabia imposes ban on international pilgrims for hajj. Politico.

[84] (2022). COVID-19: Dhs3,000 fine for not wearing facemasks in enlosed places in UAE. Gulf Today.

[85] Nasrallah T (2020). Saudi Arabia builds two makeshift hospitals in Mecca. Gulf News.

[86] (2020). Israel: New Rules Require 14 Days of Quarantine for All Persons Entering the Country. Library of Congress.

[87] Last M (2020). The first wave of COVID-19 in Israel-Initial analysis of publicly available data. PLoS One.

[88] Leshem E, Afek A, Kreiss Y (2020). Buying time with COVID-19 outbreak response, Israel. Emerg Infect Dis.

[89] Waitzberg R, Davidovitch N, Leibner G, Penn N, Brammli-Greenberg S (2020). Israel's response to the COVID-19 pandemic: tailoring measures for vulnerable cultural minority populations. Int J Equity Health.

[90] (2020). Morocco: Stepping Up to the COVID-19 Pandemic Outbreak. The World Bank.

[91] Dobrinen E, Moser L, White D, Alquwayfili S, Bingham D, Tesfai H (2023). Surveillance methods used to detect, characterize, and monitor the COVID-19 pandemic in Rocky Mountain tribal communities. Public Health Rep.

[92] Barkia A, Laamrani H, Belalia A, Benmamoun A, Khader Y (2021). Morocco's national response to the COVID-19 pandemic: public health challenges and lessons learned. JMIR Public Health Surveill.

[93] Ghobari M, El Yaakoubi A (2020). 'It is still a mystery': War-hit Yemen struggles to trace COVID-19 infection. Reuters.

[94] (2021). Yemen: Houthis Risk Civilians’ Health in Covid-19. Human Rights Watch.

[95] Muñoz-Gallego I, Meléndez Carmona MÁ, Martín Higuera C, Viedma E, Delgado R, Folgueira MD (2023). Rapid screening of SARS-CoV-2 variants, a key tool for pandemic surveillance. Sci Rep.

[96] Assiri A, Al-Tawfiq JA, Alkhalifa M, Al Duhailan H, Al Qahtani S, Dawas RA, El Seoudi AA, Alomran N, Omar OA, Alotaibi N, Almudarra SS, Alabdulkarim K, Alqahtani S, Jokhdar H (2021). Launching COVID-19 vaccination in Saudi Arabia: Lessons learned, and the way forward. Travel Med Infect Dis.

[97] Kalin S (2021). Saudi Arabia to Impose Covid-19 Vaccine Mandate. The Wall Street Journal.

[98] Barrington L, Coates S (2021). UAE mandates COVID-19 vaccines for live events. Reuters.

[99] (2022). Egypt: As Covid vaccine mandates tighten, fake vaccine certification proves rife. Middle East Eye.

[100] Fahmy S, Eltahir N, Lewis A, Heinrich M (2021). Egypt ramps up local vaccine production with eye on exports. Reuters.

[101] Abolfadl A, Mourad M, Heavens A (2021). Egypt to start local production of Sinovac vaccine mid-June- minister. Reuters.

[102] Galal B, Lazieh S, Al-Ali S, Khoshnood K (2022). Assessing vaccine hesitancy in Arab countries in the Middle East and North Africa (MENA) region: a scoping review protocol. BMJ Open.

[103] (2021). Egypt and United Arab Emirates: COVID-19 Vaccine Ruled Permissible under Islamic Law. Library of Congress.

[104] Stein R (2023). As the pandemic ebbs, an influential COVID tracker shuts down. NPR.

[105] Ritchey MD, Rosenblum HG, Del Guercio K, Humbard M, Santos S, Hall J, Chaitram J, Salerno RM (2022). COVID-19 self-test data: challenges and opportunities - United States, October 31, 2021-June 11, 2022. MMWR Morb Mortal Wkly Rep.

[106] Adambekov S, Kaiyrlykyzy A, Igissinov N, Linkov F (2016). Health challenges in Kazakhstan and Central Asia. J Epidemiol Community Health.

[107] Staadegaard L, Del Riccio M, Wiegersma S, El Guerche-Séblain C, Dueger E, Akçay M, Casalegno JS, Dückers M, Caini S, Paget J, NIC Collaborators (2023). The impact of the SARS-CoV-2 pandemic on global influenza surveillance: Insights from 18 National Influenza Centers based on a survey conducted between November 2021 and March 2022. Influenza Other Respir Viruses.

[108] Sombié I, Johnson E, Lokossou V, Amouh T, Sow A, Ogbureke N, Okolo S (2020). How does the West African Health Organisation (WAHO) contribute to the evidence based decision-making and practice during COVID-19 pandemic in ECOWAS region?. Pan Afr Med J.

[109] Balck A, Föh B, Borsche M, Rahmöller J, Vollstedt E, Waldeck F, Käding N, Twesten C, Mischnik A, Gillessen-Kaesbach G, Ehlers M, Sina C, Taube S, Busch H, Rupp J, Katalinic A, Klein C (2022). Protocol of the Luebeck longitudinal investigation of SARS-CoV-2 infection (ELISA) study - a prospective population-based cohort study. BMC Public Health.

[110] Nahari AD, Son MBF, Newburger JW, Reis BY (2022). An integrated framework for identifying clinical-laboratory indicators for novel pandemics: COVID-19 and MIS-C. NPJ Digit Med.

[111] Coccia M (2022). Improving preparedness for next pandemics: Max level of COVID-19 vaccinations without social impositions to design effective health policy and avoid flawed democracies. Environ Res.

[112] Abbott B (2023). He’s Been Naming Covid Variants. Not Everyone’s Happy. The Wall Street Journal.

[113] Katella K (2023). What to Know About EG.5 (Eris)—the Latest Coronavirus Strain. Yale Medicine.

[114] Coccia M (2020). Factors determining the diffusion of COVID-19 and suggested strategy to prevent future accelerated viral infectivity similar to COVID. Sci Total Environ.

[115] Ricoca Peixoto V, Vieira A, Aguiar P, Sentis A, Carvalho C, Rhys Thomas D, Abrantes A, Nunes C (2023). COVID-19 surveillance: Large decrease in clinical notifications and epidemiological investigation questionnaires for laboratory-confirmed cases after the 2nd epidemic wave, Portugal March 2020-July 2021. Front Public Health.

[116] Hashmi M, Beane A, Murthy S, Dondorp AM, Haniffa R, CRIT Care Asia (2020). Leveraging a cloud-based critical care registry for COVID-19 pandemic surveillance and research in low- and middle-income countries. JMIR Public Health Surveill.

