# Supplementary material for: Updated Surveillance Metrics and History of the COVID-19 Pandemic (2020-2023) in the Middle East and North Africa: Longitudinal Trend Analysis
Source: JMIR Public Health Surveill. 2024 Jun 12;10:e53219. doi: 10.2196/53219 (PMC11208839; doi:10.2196/53219)
Supplement: Multimedia Appendix 1 [file publichealth_v10i1e53219_app1.docx]

**Table S1. Static surveillance metrics for the weeks of Apr 28, 2023 (Pre-Declaration) and May 5, 2023 (Post-Declaration).**

| Country | Week | New COVID-19 cases, n | Cumulative COVID-19 cases, n | 7-day moving average of new cases | Weekly Transmission Rate per 100K Individuals | New deaths, n | Cumulative deaths, n | 7-day moving average of deaths | Death rate per 100K individuals | Conditional death rate |
| --- | --- | --- | --- | --- | --- | --- | --- | --- | --- | --- |
| Algeria | Pre | 3 | 271,745 | 6.86 | 0.01 | 0 | 6,881 | 0 | 0 | 0.03 |
|  | Post | 3 | 271,798 | 7.57 | 0.01 | 0 | 6,881 | 0 | 0 | 0.03 |
| Egypt | Pre | 0 | 516,023 | 0.57 | 0 | 0 | 24,830 | 0.29 | 0 | 0.05 |
|  | Post | 0 | 516,023 | 0 | 0 | 0 | 24,830 | 0 | 0 | 0.05 |
| Iran | Pre | 0 | 7,609,121 | 196.71 | 0 | 0 | 146,129 | 10.14 | 0 | 0.02 |
|  | Post | 0 | 7,610,333 | 173.14 | 0 | 0 | 146,188 | 8.43 | 0 | 0.02 |
| Israel | Pre | 227 | 4,823,022 | 225.71 | 2.40 | 4 | 12,508 | 2.29 | 0.04 | 0 |
|  | Post | 148 | 4,824,239 | 173.86 | 1.57 | 0 | 12,512 | 0.57 | 0 | 0 |
| Kuwait | Pre | 7 | 665,802 | 8.14 | 0 | 0 | 2,570 | 0 | 0 | 0 |
|  | Post | 5 | 665,851 | 6.29 | 0 | 0 | 2,570 | 0 | 0 | 0 |
| Lebanon | Pre | 109 | 1,237,080 | 64.14 | 1.99 | 1 | 10,905 | 0.71 | 0.02 | 0.01 |
|  | Post | 368 | 1,237,495 | 59.29 | 6.70 | 7 | 10,913 | 1.14 | 0.13 | 0.01 |
| Libya | Pre | 0 | 507,252 | 0 | 0 | 0 | 6,437 | 0 | 0 | 0.01 |
|  | Post | 0 | 507,253 | 0 | 0 | 0 | 6,437 | 0 | 0 | 0.01 |
| Morocco | Pre | 53 | 1,273,463 | 50.71 | 0 | 0 | 16,297 | 0 | 0 | 0.01 |
|  | Post | 50 | 1,273,832 | 52 | 0 | 0 | 16,297 | 0 | 0 | 0.01 |
| Oman | Pre | 0 | 399,449 | 0 | 0 | 0 | 4,628 | 0 | 0 | 0.01 |
|  | Post | 0 | 399,449 | 0 | 0 | 0 | 4,628 | 0 | 0 | 0.01 |
| Qatar | Pre | 240 | 509,440 | 445.29 | 8.90 | 0 | 690 | 0 | 0 | 0 |
|  | Post | 510,596 | 165.14 | 0 | 0 | 690 | 0 | 0 | 0 |  |
| Saudi Arabia | Pre | 121 | 841,469 | 181.29 | 0.33 | 0 | 9,646 | 0.57 | 0 | 0.01 |
|  | Post | 0 | 841,469 | 0 | 0 | 0 | 9,646 | 0 | 0 | 0.01 |
| Tunisia | Pre | 36 | 1,152,877 | 37 | 2.06 | 0 | 29,398 | 1.57 | 0.09 | 0.03 |
|  | Post | 20 | 1,153,063 | 26.86 | 1.12 | 0 | 29,407 | 1.29 | 0.06 | 0.03 |
| United Arab Emirates | Pre | 212 | 1,063,784 | 209 | 2.25 | 0 | 2,349 | 0 | 0 | 0 |
|  | Post | 172 | 1,065,156 | 196 | 1.82 | 0 | 2,349 | 0 | 0 | 0 |

Notes: “Pre” and “Post” refer to the weeks of Apr 28 and May 5, 2023, as the WHO declared the end of COVID-19 as a public health emergency of international concern on May 5, 2023. “Death rate per 100K individuals” is calculated as the ratio of “New deaths, n” over population, multiplied by 100K. “Conditional death rate” is the ratio of “Cumulative deaths, n” to “Cumulative COVID-19 cases, n.”
